# Enzymatic Hydrolysates from *Fucus vesiculosus*: Optimal Process, Chemical Profile and Bioactivity

**DOI:** 10.3390/md24070251

**Published:** 2026-07-18

**Authors:** Paulo Nova, Marta Coelho, Sara A. Cunha, Manuela Machado, Ana R. Costa-Pinto, Ana Maria Gomes

**Affiliations:** 1 CBQF—Centro de Biotecnologia e Química Fina Laboratório Associado, Escola Superior de Biotecnologia, Universidade Católica Portuguesa, Rua Diogo Botelho 1327, 4169-005 Porto, Portugal; pnova@ucp.pt (P.N.); mcoelho@ucp.pt (M.C.); scunha@ucp.pt (S.A.C.); mmachado@ucp.pt (M.M.); amgomes@ucp.pt (A.M.G.); 2 i3S—Instituto de Investigação e Inovação em Saúde, Universidade do Porto, 4200-319 Porto, Portugal; 3 IPATIMUP—Instituto de Patologia Molecular e Imunologia da Universidade do Porto, 4200-135 Porto, Portugal

**Keywords:** macroalgae, extraction, carbohydrases, proteases, enzyme-assisted extraction, value added compounds

## Abstract

*Fucus vesiculosus* (FV) is a brown macroalga rich in bioactive compounds with significant industrial potential. This study aimed to produce enzyme-assisted water-soluble hydrolysates from FV with optimized antioxidant activity using Box–Behnken experimental designs. Two extraction methods were evaluated: cellulase alone (FVc) and a combination of cellulase and alcalase (FVca). The optimization focused on enzyme concentration, temperature, and incubation time, measuring extraction yield, total phenolic content as determined by the Folin–Ciocalteu assay (non-specific reducing capacity index, FC-derived TPC), total antioxidant capacity (TAC), and oxygen radical absorbance capacity (ORAC). Results demonstrated that the combined dual-enzyme (FVca) treatment was highly efficient, simultaneously maximizing the extraction yield (39.41%) and the overall reducing capacity (TAC of 142.80 µmol TE/g, ORAC of 477.64 µmol TE/g, and a FC-derived TPC of 252.57 mg GAE/g). Due to its higher potential, FVca was further characterized, revealing a rich profile of essential amino acids, low-molecular-weight peptides, and a diverse phenolic profile, dominated by phloroglucinol (6.23 mg/g). In addition, the FVca hydrolysate demonstrated severe abiotic interference with the redox viability assay at 10 mg/mL in Caco-2 human colorectal adenocarcinoma cell cultures. These findings highlight that combining cellulase and alcalase effectively solubilizes key bioactive compounds, yielding a hydrolysate highly promising for industrial and biotechnological applications.

## 1. Introduction

Algae are photosynthetic eukaryotes with a rich evolutionary history and a crucial role in ecosystems, contributing, together with other marine phototrophs, to approximately 50% of all oxygen produced [1,2]. These organisms have diverse biology and can adapt and grow in harsh environmental conditions such as temperature, sunlight, salinity, and carbon dioxide supply (CO_2_). During this process, they produce highly valuable bioactive molecules like polysaccharides, proteins, minerals, glycoproteins, and other secondary metabolites with antitumor, antioxidant, anti-inflammatory, anticoagulant, and antibacterial activities [3,4,5]. Consumers’ and industries’ growing interest in seaweeds led global production to reach a record 37.8 million tons in 2022, continuing its upward trajectory from 35.2 million tons in 2021 [6]. This represents a more than three-fold increase from the 11.8 million tons produced in 2001. China remains the undisputed leader in worldwide seaweed cultivation, consistently driving the vast majority of global aquaculture output [6].

*Fucus vesiculosus* (*F. vesiculosus*) is a brown macroalga (*Phaeophyceae*) spread all over the world in the cold waters of rocky intertidal habitats, with a prevalence in the northeast Pacific and North Atlantic coastline [7,8]. This macroalga is very common in Portuguese seawaters and has a notable nutritional profile along with functional properties, which contribute to its current use as a valued food ingredient [9,10]. *Fucus vesiculosus* contains a high protein content, along with mono- and polyunsaturated fatty acids, vitamins, and minerals such as magnesium, calcium, and iron [10]. The main dietary fibers of this macroalga/seaweed are cellulose, fucans, alginates, and laminarans, with alginic acid representing the predominant polysaccharide (up to 59% dry weight) [10]. Moreover, it presents phlorotannins such as fucols and fucophlorethols [11]. *Fucus vesiculosus* is a rich reservoir of bioactive compounds that exhibit antitumor, antioxidant, anti-inflammatory and antidiabetic activities and, as such, is of high interest for the biotechnological, medical and food industries [3,11,12,13,14,15].

Extraction is the most suitable method for obtaining these bioactive compounds, prompting growing research efforts toward the development of sustainable extraction systems [9,13,16,17,18]. Among the available green extraction technologies, enzymatic extraction stands out for its eco-friendliness (it does not require harmful chemicals or organic solvents), high efficiency, scalability, and its ability to convert water-insoluble materials into water-soluble ones [16,19]. This method uses hydrolases such as carbohydrases and proteases to break down the complex cell walls of macroalgae and release the compounds of interest [16,19]. To enhance extraction efficiency, it is essential to consider factors such as pH, substrate ratio (solvent to enzyme), temperature, and mixing conditions [16,19,20].

In previous work, we studied the chemical composition and antioxidant potential of *F. vesiculosus* for food, health, and biotechnological applications [10,16]. Building on these findings, the present study focuses on the production of enzymatically assisted water-soluble hydrolysates rich in antioxidants and previously unreported compounds from *F. vesiculosus*, alongside optimized macroalgal hydrolysates. Through analytical characterization and cytotoxic assessment, we evaluate these bioactive profiles and highlight critical assay interferences that must be accounted for in the future therapeutic development of these extracts, including anticarcinogenic applications.

## 2. Results

### 2.1. Optimization of the Production of Fucus vesiculosus Hydrolysates

In previous works, we characterized the composition of macroalga *F. vesiculosus*, revealing an interesting nutritional and bioactive profile, including protein, polysaccharide, fat, carbohydrate, and dietary fiber contents, as well as bioactive properties [10,16]. Furthermore, we tested the effects of different enzyme-assisted extractions on *F. vesiculosus* using either isolated or combined alcalase^®^, cellulase^®^, or Viscozyme treatments [16]. These preliminary studies showed that the use of cellulase^®^ alone and the sequential use of cellulase^®^ + alcalase^®^ are the most suitable strategies for extracting valuable compounds from *F. vesiculosus* [16]. To maximize the extraction of valuable compounds from this brown macroalga, a Box–Behnken experimental design was employed. The design resulted in 15 experimental runs for *F. vesiculosus* extracted with cellulase^®^ and 27 experimental runs for *F. vesiculosus* extracted with cellulase^®^ + alcalase^®^ combinations. The DOE matrix and results for the dependent variables of *F. vesiculosus* extracted with cellulase^®^ are presented in Table 1.

**Figure 1:**
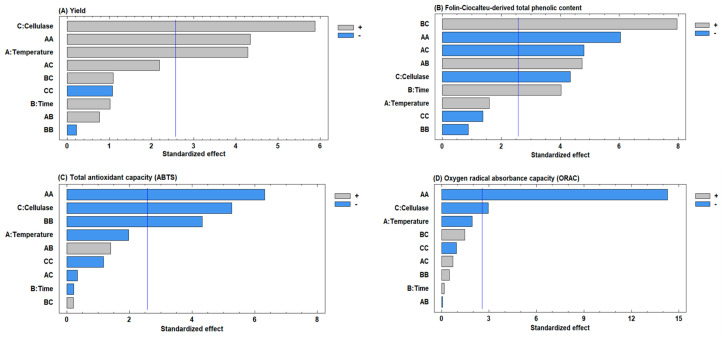
Recalculated Pareto charts showing the effects of three experimental factors on FV extraction using cellulase. Charts are presented in decreasing order of effect for yield (**A**), Folin–Ciocalteu-derived total phenolic content (**B**), total antioxidant capacity (**C**) and oxygen radical absorbance capacity (**D**). Vertical reference lines represent the significance level (*p* = 0.05).

**Figure 2:**
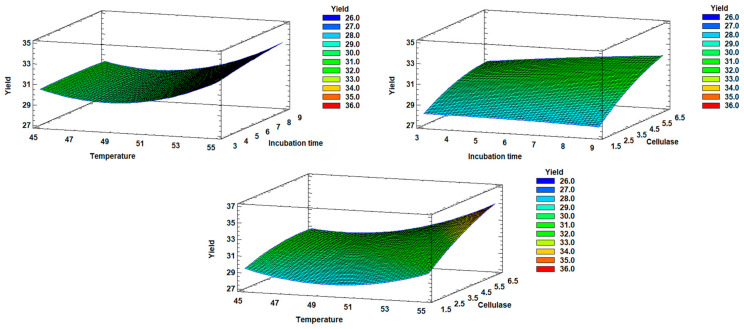
Yield response surface plots for FV extraction using cellulase. Each surface corresponds to the combined effect of two experimental factors on yield (%), with the remaining factor fixed at its central level.

**Figure 3:**
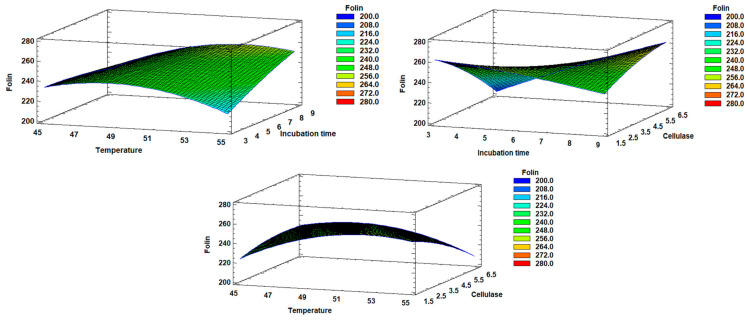
Folin–Ciocalteu-derived total phenolic content (non-specific reducing capacity index) response surface plots for FV extraction using cellulase. Each surface corresponds to the combined effect of two experimental factors on Folin–Ciocalteu-derived total phenolic content (mg GAE/g dry extract), with the remaining factor fixed at its central level.

**Figure 4:**
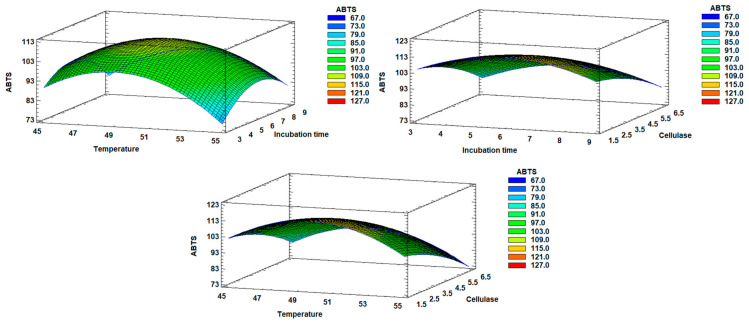
Total antioxidant capacity (ABTS) response surface plots for FV extraction using cellulase. Each surface corresponds to the combined effect of two experimental factors on ABTS (µmol TE/g dry extract), with the remaining factor fixed at its central level.

**Figure 5:**
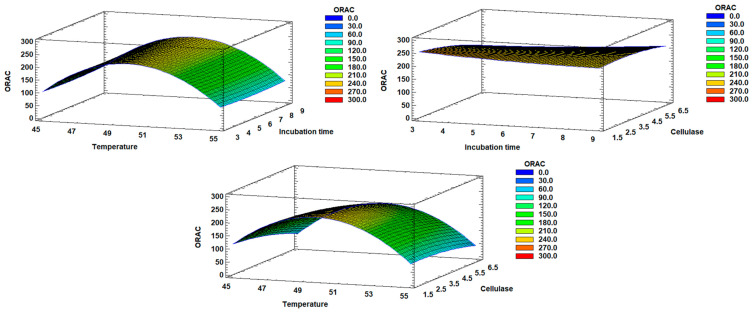
Oxygen radical absorbance capacity (ORAC)) response surface plots for FV extraction using cellulase. Each surface corresponds to the combined effect of two experimental factors on ORAC (µmol TE/g dry extract), with the remaining factor fixed at its central level.

As shown in Table 1, the highest hydrolysate yields were obtained in runs 2, 10, and 11 (32.90%, 32.80%, and 34.82%, respectively), all obtained at a hydrolysis temperature of 55 °C. The highest total antioxidant capacity (ABTS) values were observed for runs 4, 9 and 15, which were performed at central or lower cellulase percentages, yielding 109.47, 109.50 and 110.82 µmol TE/g dry extract, respectively. For the non-specific reducing capacity index Folin–Ciocalteu-derived total phenolic content (FC-derived TPC), the highest values were obtained for runs 4, 5, and 9 (262.98, 259.55, and 253.88 mg GAE/g dry extract, respectively), all conducted at the central hydrolysis temperature (50 °C), with varying cellulase levels and incubation times. A similar temperature-dependent trend was observed for oxygen radical absorbance capacity (ORAC), with the highest values achieved in runs 4, 7, and 15 (262.63, 243.10, and 232.37 µmol TE/g dry extract, respectively), also at lower or central cellulase levels. To facilitate interpretation of DOE results, graphical analyses were employed. Pareto charts (Figure 1) were used to identify factors with significant effects on each response, whereas response surface plots are presented in Figure 2, Figure 3, Figure 4 and Figure 5. Detailed ANOVA tables for each response are provided in the Supplementary Materials (Tables S1–S4).

Yield values obtained across the experimental runs ranged from 27.83 to 34.82%. The recalculated Pareto chart showed that cellulase percentage (C) and the quadratic effect of temperature (A^2^) were the most significant factors influencing yield. The final adjusted regression model for yield is presented in Equation (1). As shown in Table 2, the adjusted model explained 83.8% of the variability in yield percentage (R^2^ = 0.838), indicating adequate fit to the experimental data. Table S1 provides a detailed ANOVA for yield response.

Yield = 201.991 − 6.85042A − 0.961806B − 2.66247C + 0.0669167A^2^ + 0.0188333AB + 0.072AC − 0.00967593B^2^ + 0.06BC − 0.081893C^2^ (1)

where A represents temperature, B incubation time and C cellulase percentage.

Folin–Ciocalteu-derived total phenolic content values ranged from 208.79 to 262.98 mg GAE/g dry extract. The recalculated Pareto chart showed that the interaction between incubation time and cellulase percentage (BC) was the most significant factor influencing the FC-derived TPC positively. Additional relevant effects included the quadratic effect of temperature (A^2^), and its interactions with incubation time (AB) and cellulase percentage (AC). The final adjusted regression model for FC-derived TPC is presented in Equation (2). As shown in Table 2, the adjusted model explained 92.6% of the variability in FC-derived TPC (R^2^ = 0.926) indicating a strong fit to the experimental data. Table S2 provides a detailed ANOVA for FC-derived TPC response.

Folin–Ciocalteu-derived total phenolic content = −1159.46 + 58.161A − 41.85B + 34.9101C − 0.583183A^2^ + 0.732833AB − 0.990444AC − 0.237176B^2^ + 2.73519BC − 0.661646C^2^ (2)

Total antioxidant capacity values ranged from 73.60 to 110.82 µmol TE/g dry extract. The recalculated Pareto chart showed that the quadratic effect of temperature (A^2^), cellulase percentage (C) and the quadratic effect of incubation time (B^2^) were the most significant factors affecting antioxidant activity measured via the ABTS method (electron transfer-based). The final adjusted regression model for total antioxidant capacity is presented in Equation (3). As shown in Table 2, the adjusted model explained 85.0% of the variability in the response (R^2^ = 0.850) indicating a satisfactory fit to the experimental data. Table S3 provides a detailed ANOVA for total antioxidant capacity response.

Total antioxidant capacity = −1432.7 + 62.0542A + 2.83611B + 3.5821C − 0.638167A^2^ + 0.226667AB − 0.0733333AC − 1.21435B^2^ + 0.0740741BC − 0.583539C^2^ (3)

where A represents temperature, B incubation time and C cellulase percentage.

Oxygen radical absorbance capacity (ORAC) values ranged from 70.15 to 262.63 µmol TE/g dry extract. The recalculated Pareto chart revealed that the quadratic effect of temperature (A^2^) was the most significant factor influencing antioxidant capacity measured by ORAC (hydrogen atom transfer-based) exhibiting the highest standardized effect. In addition, temperature (A) and cellulase percentage (C) also showed significant effects on this response. The final adjusted regression model for ORAC is presented in Equation (4). As shown in Table 2, the adjusted model explained 93.8% of the variability in ORAC values (R^2^ = 0.938) indicating an excellent fit to the experimental data. Table S4 provides a detailed ANOVA for ORAC response.

ORAC = −13258.4 + 546.646A − 11.4181B − 37.3173C − 5.51183A^2^ − 0.0416667AB + 0.6AC + 0.522685B^2^ + 2.02222BC − 1.79177C^2^ (4)

where A represents temperature, B incubation time and C cellulase percentage.

**Figure 6:**
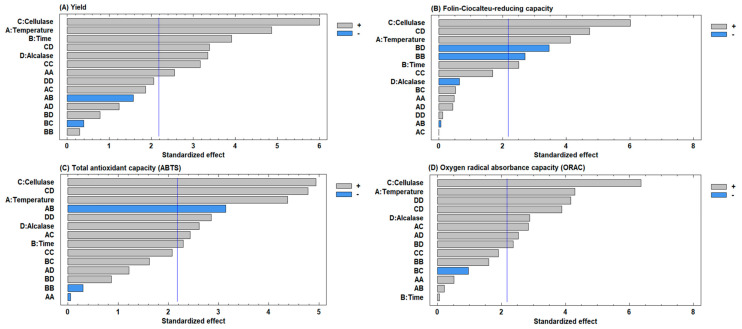
Recalculated Pareto charts showing the effects of four experimental factors on FV extraction using cellulase and alcalase. Charts are presented in decreasing order of effect for yield (**A**), Folin–Ciocalteu-derived total phenolic content (**B**), total antioxidant capacity (**C**) and oxygen radical absorbance capacity (**D**). Vertical reference lines represent the significance level (*p* = 0.05).

**Figure 7:**
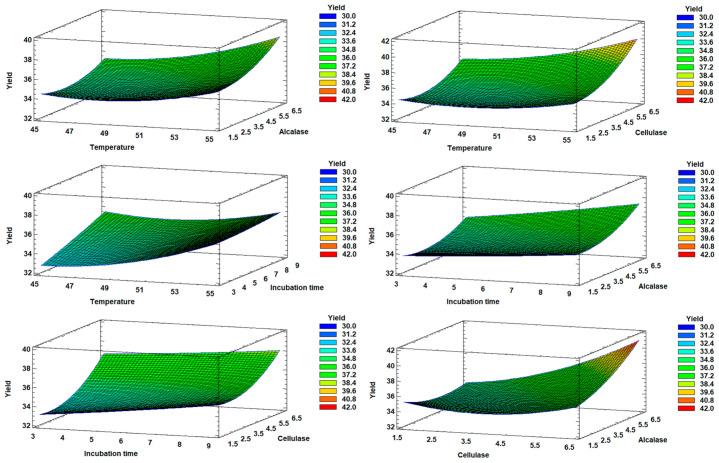
Yield response surface plots for FV extraction using cellulase and alcalase. Each surface corresponds to the combined effect of two experimental factors on yield (%), with the remaining factors fixed at its central level.

**Figure 8:**
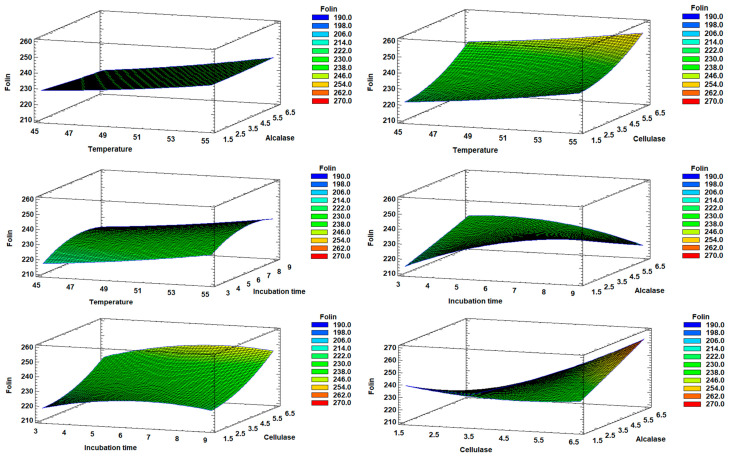
Folin–Ciocalteu-derived total phenolic content surface plots for FV extraction using cellulase and alcalase. Each surface corresponds to the combined effect of two experimental factors on Folin–Ciocalteu-derived total phenolic content (mg GAE/g dry extract), with the remaining factors fixed at its central level.

**Figure 9:**
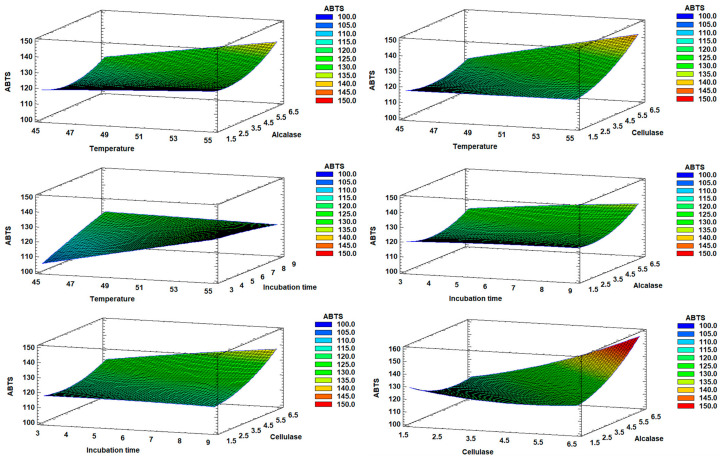
Total antioxidant capacity (ABTS) response surface plots for FV extraction using cellulase and alcalase. Each surface corresponds to the combined effect of two experimental factors on ABTS (µmol TE/g dry extract), with the remaining factors fixed at its central level.

**Figure 10:**
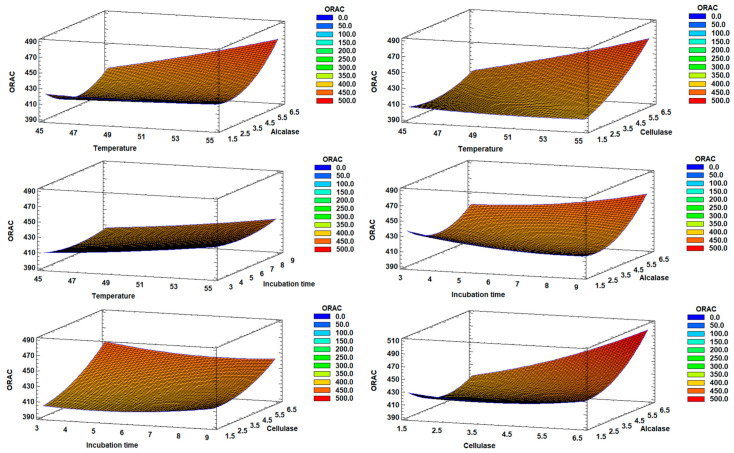
Oxygen radical absorbance capacity (ORAC) response surface plots for FV extraction using cellulase and alcalase. Each surface corresponds to the combined effect of two experimental factors on ORAC (µmol TE/g dry extract), with the remaining factors fixed at its central level.

Regarding *F. vesiculosus* extraction using a combined enzyme-assisted hydrolysis with cellulase and alcalase, the highest hydrolysate yields were obtained in runs 10, 14, and 21, corresponding to 39.24%, 38.70%, and 38.35%, respectively. The maximum yield was obtained when both cellulase and alcalase were applied at their maximum percentage levels (6%). In contrast, the second and third highest yields were achieved when one enzyme was set at its maximum percentage level (6%) while the other was kept at the central percentage level (3.75%). The top three highest total antioxidant capacity values were observed in runs 10, 21, and 18 (145.4, 138.6, and 135.9 µmol TE/g dry extract, respectively). These runs were characterized by having the cellulase percentage set at the maximum level (6%). For the Folin–Ciocalteu-derived total phenolic content, the highest values were obtained for the exact same runs—10, 21, and 18 (252.38, 247.86, and 247.61 mg GAE/g dry extract, respectively). The maximum value for both assays (run 10) was obtained under central conditions for temperature and incubation time (50 °C and 6 h), but maximum conditions for both enzymes (6% cellulase and 6% protease). Oxygen radical absorbance capacity (ORAC) reached its highest levels in runs 10, 14, and 21 (475.2, 463.9, and 454.6 µmol TE/g dry extract, respectively). These optimum ORAC conditions were characterized by central to high levels of temperature (50–55 °C), a central incubation time of 6 h, and at least one enzyme percentage set at the maximum level (6%). A complete overview of the experimental results is presented in Table 3. For improved interpretation of the effects of the experimental variables, DOE results were further analyzed using Pareto charts (Figure 6), which identify the most significant coefficients for each response. The corresponding response surface plots are shown in Figure 6, Figure 7, Figure 8, Figure 9 and Figure 10. Detailed ANOVA tables for each response are provided in the Supplementary Materials (Tables S5–S8). Table 4 presents the summary of model fit statistics for the studied responses.

For the yield (%), *F. vesiculosus* hydrolysis with cellulase and alcalase yielded extracts with a yield in the range of 32.92% to 39.24%. The recalculated Pareto charts showed that cellulase has the highest positive standardized effect, indicating it is the most significant factor positively influencing yield. Furthermore, temperature and incubation time also have strong positive effects on this parameter, though less significant than cellulase. Alcalase has a smaller, yet still significant, positive effect. Regarding interactions between the analyzed parameters cellulase × alcalase (CD), quadratic effect of cellulase (CC), quadratic effect of temperature (AA) and quadratic effect of alcalase (DD), the Pareto charts demonstrated that these interactions also have positive effects on the obtention of higher yields. On the contrary, temperature × time (AB) interaction is the most significant negative effect leading to reduced yield.

The final adjusted model is presented in Equation (5). Table S5 provides a detailed ANOVA for yield response.

Yield = 117.071 − 3.21017A + 2.01139B − 4.79753C − 3.87901D + 0.0326833A^2^ − 0.0388333AB + 0.0613333AC + 0.0406667AD + 0.0106481B^2^ − 0.0214815BC + 0.042963BD + 0.200165C^2^ + 0.247407CD + 0.130288D^2^ (5)

where A represents temperature, B incubation time, C cellulase percentage and D alcalase percentage.

Regarding the FC-derived TPC, *F. vesiculosus* extracted with an enzyme-assisted combination of cellulase and alcalase showed values in the range of 214.75 to 252.38 mg GAE/g dry extract. The recalculated Pareto charts showed that the enzyme cellulase (C) is the most influential factor for increasing FC-derived TPC, showing the strongest positive effect among all variables. Furthermore, the interaction between cellulase and alcalase (CD) also shows a significant positive effect, indicating synergism when both enzymes are combined. Temperature (A) also positively influences FC-derived TPC, as the Pareto chart shows that controlled heat application supports higher phenolic content. Incubation time (B) has a modest positive effect on its own. The BD (Time × Alcalase) and the quadratic effect of time, BB (Time × Time), interactions exhibit strong negative effects, suggesting that prolonged processing or specific combinations of time and alcalase reduce phenolic content. The final adjusted model is presented in Equation (6). Table S6 provides a detailed ANOVA for the Folin–Ciocalteu-derived total phenolic content response.

Folin–Ciocalteu-derived total phenolic content = 265.168 − 3.19383A+ 13.3819B − 11.2531C − 6.59753D + 0.0407333A^2^ − 0.0105AB − 0.000666667AC + 0.0953333AD − 0.638935B^2^ + 0.192593BC − 1.26037BD + 0.707078C^2^ + 2.29778CD + 0.0490535D^2^ (6)

where A represents temperature, B incubation time, C cellulase percentage and D alcalase percentage.

For total antioxidant capacity, *F. vesiculosus* extracted with an enzyme-assisted combination of cellulase and alcalase registered values in a range of 106.1 to 145.4 µmol TE/g dry extract. The recalculated Pareto charts indicated that cellulase (C) is the most influential factor, exhibiting a strong positive effect on antioxidant capacity. The interaction between cellulase and alcalase (CD) also has a significant positive effect, indicating a synergistic benefit when used together. Temperature (A) has a notable positive influence on its own; however, its interaction with incubation time (AB) exerts the strongest negative effect on ABTS capacity. Alcalase (D) contributes positively on its own, but its self-interaction (DD) negatively impacts antioxidant capacity. The final adjusted model is presented in Equation (7). Table S7 shows that the adjusted model explains 79.2% of total antioxidant capacity variability (R2). Table S7 provides a detailed ANOVA for total antioxidant capacity response.

ABTS = 101.217 + 1.42667A + 19.5B − 33.9185C − 25.0593D − 0.0035A^2^ − 0.413333AB + 0.426667AC + 0.213333AD − 0.0569444B^2^ + 0.474074BC + 0.251852BD + 0.701235C^2^ + 1.86173CD + 0.962963D^2^ (7)

where A represents temperature, B incubation time, C cellulase percentage and D alcalase percentage.

For oxygen radical absorbance capacity (ORAC), *F. vesiculosus* extracted with cellulase and alcalase showed a profile ranging from 394.8 to 475.2 µmol TE/g dry extract. The recalculated Pareto charts showed that cellulase (C) has the most significant positive effect on ORAC, making it the key driver in enhancing antioxidant capacity. Temperature (A) also presents a notable positive role, contributing strongly to ORAC improvement. The other enzyme, alcalase (D), has a moderate but significant positive impact on this parameter. Furthermore, the interaction between cellulase and alcalase (CD) and the quadratic effect of alcalase (DD) are highly beneficial. However, the BC (Temperature × Time) interaction indicates a negative effect. The final adjusted model is presented in Equation (8). Table S8 provides a detailed ANOVA for oxygen radical absorbance capacity response.

ORAC = 1028.5 − 13.9067A − 14.6833B − 68.1506C − 91.7506D + 0.0781667A^2^ + 0.0633333AB + 1.11333AC + 0.991111AD + 0.678241B^2^ − 0.62963BC + 1.54444BD + 1.43786C^2^ + 3.37778CD + 3.13663D^2^ (8)

where A represents temperature, B incubation time, C cellulase percentage and D alcalase percentage.

### 2.2. F. vesiculosus Hydrolysate Single and Multiple Response Optimization

Both macroalgae hydrolysates were optimized to maximize yield (%) and the release of antioxidant-active compounds. A multi-response optimization was performed using Derringer desirability analysis to maximize the overall desirability of the studied responses [21,22]. For *F. vesiculosus* extracted with cellulase, a desirability value of 0.67 was obtained, and the predicted optimal hydrolysis conditions were: 4.5% cellulase, 51 °C and an incubation time of 7.8 h. Under these conditions, the model predicted a yield of 31.02%, a Folin–Ciocalteu-derived total phenolic content of 252.8 mg GAE/g dry extract, a total antioxidant capacity of 100.9 µmol TE/g dry extract, and an ORAC value of 216.1 µmol TE/g dry extract. The optimal conditions predicted for single and multiple response optimization are presented in Table 5.

For *F. vesiculosus* extracted using the combined action of cellulase and alcalase, a desirability value of 1.0 was obtained, and the predicted optimal hydrolysis conditions were: 5.97% cellulase, 5.95% alcalase, a hydrolysis temperature of 53.8 °C, and an incubation time of 5 h. Under these conditions, the predicted responses were a yield of 40.6%, a Folin–Ciocalteu-derived total phenolic content of 256.0 mg GAE/g dry extract, a total antioxidant capacity of 149.0 µmol TE/g dry extract, and an ORAC value of 487.2 µmol TE/g dry extract. The predicted optimal conditions and corresponding responses are presented in Table 6.

To better understand the relationship between the evaluated parameters in the dual-enzyme design, a Pearson correlation analysis was performed (Table 7). The analysis revealed positive correlations across all four responses. The gravimetric yield exhibited a significant positive correlation with ABTS (*r* = 0.852), ORAC (*r* = 0.749), and FC-derived TPC (*r* = 0.745). Similarly, the antioxidant assays and the non-specific reducing capacity index (FC-derived TPC) were also positively correlated with one another, ranging from moderate (*r* = 0.519 for ORAC vs. FC-derived TPC) to strong (*r* = 0.746 for ORAC vs. ABTS).

### 2.3. Bioactive Properties, Chemical Profile and Cytotoxicity of Fucus vesiculosus Hydrolysates Obtained by Combined Enzymatic Hydrolysis

Hydrolysates of *Fucus vesiculosus* obtained using the combined action of cellulase and alcalase showed improved performance across all evaluated parameters compared to hydrolysis with cellulase alone. Therefore, this combined methodology was chosen for further study. The optimized extract/hydrolysate was subsequently produced and analyzed, and the experimental results confirmed the predictions of the DOE model. The results are presented in Table 8.

The chemical profile of *F. vesiculosus* hydrolysates obtained using the combined action of cellulase and alcalase (FVca) was analyzed in terms of amino acids, peptides and phenolics as well as cytotoxicity. Regarding amino acid composition, the FVca hydrolysate showed a rich profile, with a total of 14 amino acids detected. It was particularly rich in Glx (glutamic acid and glutamine, 27.73 mg/g) and Asx (aspartic acid and asparagine, 10.72 mg/g). Six essential amino acids—histidine, isoleucine, leucine, methionine, phenylalanine, and threonine—were detected. The complete amino acid profile is presented in Table 9.

**Figure 11:**
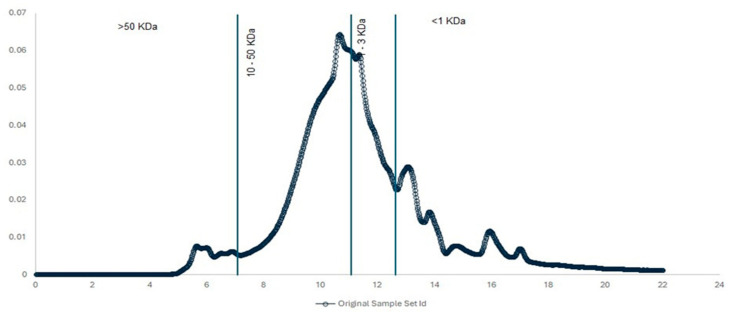
Peptide profile of *F. vesiculosus* extracted with cellulase and alcalase combination.

The chromatographic profiles of FVca hydrolysates obtained using the combined action of cellulase and alcalase are presented in Figure 11. The chromatograms reveal clear differences in protein size distribution within the hydrolysate, as evidenced by variations in absorbance peaks across different molecular weight ranges. These ranges were classified into six categories: >50 kDa, 10–50 kDa, 5–10 kDa, 3–5 kDa, 1–3 kDa, and <1 kDa, each reflecting differences in peptide composition. Pronounced peaks were observed in the lower molecular weight ranges (5–10 kDa and 3–5 kDa) indicating the presence of smaller peptides and protein fragments. The occurrence of multiple peaks within these fractions suggests a complex mixture of low-molecular-weight peptides, which may be associated with potential biological activities. Overall, the chromatographic profile demonstrates abundant and diverse peptides, supporting the potential of this hydrolysate as a rich source of bioactive peptides. The complete peptide profile is shown in Figure 11.

The phenolic profile of FVca hydrolysate obtained via the combined enzymatic action of cellulase and alcalase is summarized in Table 10. HPLC analysis resolved a total of nine distinct peaks. Based on comparisons of retention times and UV-Vis spectral data with those of standard compounds, six phenolic compounds were positively identified: phloroglucinol, gallic acid, epigallocatechin, protocatechuic acid, tiliroside, and p-coumaric acid.

**Figure 12:**
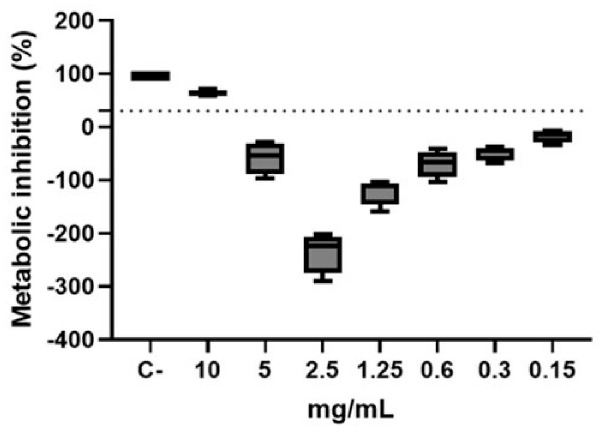
Metabolic inhibition of Caco-2 Cells treated with *F. vesiculosus* extracted with cellulase and alcalase. Note: the sign convention for the *y*-axis is such that positive values denote metabolic inhibition (toxicity), whereas negative values indicate an increase in cellular metabolic activity or proliferation relative to the control.

The FVca hydrolysate exhibited a biphasic effect on Caco-2 cell metabolic activity (Figure 12). Cytotoxic effects exceeding the ISO 10993-5 threshold (>30% inhibition) were only observed at the highest tested concentration of 10 mg/mL, which resulted in approximately 60% metabolic inhibition. Conversely, at concentrations ≤5 mg/mL (from 0.15 to 5 mg/mL), the hydrolysate induced negative inhibition values, peaking at −250% at 2.5 mg/mL. This indicates an increase in the fluorescent signal relative to the untreated control.

## 3. Discussion

*Fucus vesiculosus* presents a valuable biochemical profile and has attracted increasing interest for applications in the biomedical, food, and cosmetic industries [3,9,13,23,24]. However, the efficient release of these bioactive compounds remains technologically challenging due to the structural complexity of the macroalgal cell wall rich in cellulose, alginate, and fucoidan [25]. In this context, the application of Box–Behnken experimental designs enabled a systematic optimization of enzyme-assisted hydrolysis conditions while accounting for nonlinear effects and factor interactions [16]. The Box–Behnken Design of Experiments offers several advantages, enabling us to build robust quadratic regression models, quantify interactions among process variables, and achieve higher optimization accuracy with fewer experimental runs than factorial designs [26,27]. Beyond reducing experimental effort, this multivariate approach provided insights into the relative contribution of processing variables and their synergistic behavior, which cannot be captured through one factor-at-a-time strategies. Similar DOE frameworks have been used effectively in micro- and macroalgae processing for mercury removal by the macroalgae *Ulva* sp., cobalt biosorption by macroalgae *Padina pavonica*, fucoidan degradation and antioxidant activity of ultrasound-assisted extraction of *F. vesiculosus*, carotenoid extraction from *Isochrysis galbana* and optimization of peptide-rich bioactive hydrolysates from the microalgae *Chlorella vulgaris* [28,29,30,31,32].

For all tested responses, *F. vesiculosus* hydrolysates obtained via the combined action of cellulase and alcalase consistently outperformed those produced with cellulase alone, highlighting a good synergistic effect between the two enzymes. This synergy is likely due to the complementary modes of action of each enzyme: while cellulase breaks down the cellulose and other polysaccharides present in *F. vesiculosus* cell walls, alcalase hydrolyzes proteins embedded in or linked to cell wall structures, thereby increasing cell wall permeability and facilitating the release of antioxidant compounds [33,34,35,36]. Importantly, the DOE analysis revealed that this synergy is strongly modulated by temperature and enzyme percentage, with quadratic effects indicating that excessive variable levels can negatively affect response outcomes, likely due to thermal degradation or enzyme deactivation. In fact, the combined analysis of Folin–Ciocalteu-derived total phenolic content, a non-specific reducing capacity index, ABTS, and ORAC responses highlights the central role of temperature-dependent effects and enzyme-assisted release mechanisms in determining the antioxidant profile of *F. vesiculosus* hydrolysates. The strong influence of temperature, particularly through quadratic effects observed across all three responses, suggests the existence of a narrow thermal window that favors polyphenol release while limiting thermal degradation. This behavior is consistent with the thermal sensitivity of phenolic compounds and enzymatic systems in brown macroalgae [16]. The significant interaction between incubation time and cellulase concentration observed for FC-derived TPC indicates that extended enzymatic action enhances the release of compounds with reducing capacity, including phenolic compounds, which contributes, at least in part, to the antioxidant capacity measured by both ABTS and ORAC assays. However, the differential sensitivity of ABTS and ORAC to processing conditions reflects their distinct reaction mechanisms, namely electron-transfer versus hydrogen-atom transfer, respectively, suggesting that not all extracted phenolics contribute equally to each antioxidant pathway. In particular, the greater sensitivity of ORAC to temperature variations indicates that compounds responsible for such radical scavenging may be more susceptible to thermal degradation or structural modification. It is important to note that, although the Folin–Ciocalteu assay is widely employed to estimate total phenolic content, it reflects the overall reducing capacity of a sample and may also respond to non-phenolic reducing constituents. Accordingly, low-molecular-weight peptides, amino acids, and other redox-active molecules generated or released during hydrolysis may have contributed to the measured FC response. Overall, these results demonstrate that the antioxidant capacity of *F. vesiculosus* hydrolysates is governed not only by the abundance of phenolic compounds but also by the qualitative composition and stability of both phenolic and non-phenolic antioxidants. These findings emphasize that maximizing extraction efficiency in macroalgae and associated functional bioactivity is not solely a function of enzyme load but rather of balanced process conditions that preserve bioactivity while enabling effective matrix disruption. Furthermore, the strong positive correlations observed among all responses provide critical context for the multi-response optimization results. Unlike the single-enzyme design, where competing parameters required a clear trade-off, the dual-enzyme responses are highly collinear. This collinearity explains the optimization desirability score of 1.0, as all four parameters co-maximize toward the high-enzyme boundary (6% cellulase and 6% alcalase).

The enzymatic hydrolysis of *F. vesiculosus* using cellulase and alcalase proved effective at releasing antioxidant compounds, yielding an ORAC value of 477 µmol TE/g. This value is competitive when compared to those of other marine seaweeds. For instance, the red macroalga *Asparagopsis armata* exhibited a similar maximum ORAC of 465.18 µmol TE/g, but only after specific solvent fractionation [37]. Furthermore, our enzyme-assisted extract outperformed several microalgal species. Studies on raw marine and freshwater microalgae, such as *Nannochloropsis granulata*, *Neochloris oleoabundans*, and *Scenedesmus obliquus*, reported much lower ORAC values ranging from 44.06 to 69.48 µmol TE/g of algal biomass [38].

To further elucidate the molecular basis underlying the enhanced antioxidant responses observed under optimized conditions, a phenolic profiling was performed on the optimal *F. vesiculosus* hydrolysate using the combined action of cellulase and alcalase at optimum conditions (FVca hydrolysate). The successful quantification of phenolic compounds, particularly the high content of phloroglucinol (6.23 mg/g), indicates that the combination of cellulase and alcalase is effective in releasing intracellular phenolics from *F. vesiculosus* [39]. As previously mentioned, cellulase likely facilitates the hydrolysis of the polysaccharide-rich algal cell wall (including cellulose and fucoidan), while alcalase degrades membrane proteins, releasing phenolic compounds that are typically bound to the cell wall matrix [14,39]. This dual enzymatic action allows for the extraction of phenolics that might otherwise remain inaccessible when using conventional solvent-based extraction methods, which have been shown to yield significantly lower total phenolic contents [16]. Consistent with these observations, Nguyen et al. (2024) demonstrated that alcalase-assisted extraction significantly enhanced phenolic recovery from brown algae compared to water or ethanol extraction alone, validating the relevance of protease treatments for complex algal matrices [40]. A notable observation of the chromatographic profile is the presence of significant unidentified peaks, specifically the late-eluting compound at 58.00 min. Given the chemical characteristics of *Fucus* species, this compound likely corresponds to a high-molecular-weight phlorotannin or a complex flavonoid-derived structure [41]. These unidentified fractions may also represent sulfated phenolic conjugates or phlorotannin oligomers, which have recently been reported in *Fucus* spp. using high-resolution mass spectrometry [23,42]. The absorption maximum at 346 nm, characteristic of the B-ring absorption band (Band I) of flavonoids, further supports the hypothesis that this compound may be a flavonoid conjugate that retains structural integrity under the applied enzymatic hydrolysis conditions [43]. While HPLC-DAD allowed for the quantification of specific low-molecular-weight phenolics, cumulative concentration of phenolic compounds was lower than FC-derived TPC values. This discrepancy is not unexpected, as the two methods evaluate different analytical endpoints. HPLC selectively quantifies targeted phenolic compounds, whereas the Folin–Ciocalteu assay measures the reducing capacity of the sample and is known to respond to a broader range of redox-active constituents. Given the presence of amino acids and low-molecular-weight peptides identified in FVca, these compounds may have contributed to the FC response, thereby increasing the apparent TPC. Furthermore, the HPLC analysis may not capture the full diversity of phenolic constituents present in the extract. Indeed, several major peaks (notably at 58.00 min) remain unidentified. To address this, our research group intends to employ Liquid Chromatography-Time-of-Flight Mass Spectrometry (LC-MS TOF) in upcoming studies to definitively elucidate the structure of these unknown compounds and confirm the presence of high-molecular-weight phlorotannins. The FVca hydrolysate presented a rich amino acid profile comprising six essential amino acids (histidine, isoleucine, leucine, methionine, phenylalanine, and threonine) as well as substantial levels of Glx (glutamic acid and glutamine, 27.73 mg/g) and Asx (aspartic acid and asparagine, 10.72 mg/g). Essential amino acids must be supplied through the diet, as the human body is incapable of synthesizing them on its own, and they play critical roles in immunity, metabolism, neurotransmission, and muscle protein synthesis [44,45,46]. Furthermore, the Glx and Asx pools, representing the main amino acid constituents of the FVca hydrolysate, play crucial roles in human metabolism. Glx, acting as a dynamic interconvertible pool of glutamic acid and glutamine in vivo, is vital for gut health, muscle repair, immune function, and brain activity [47,48,49]. Glutamine, acting from this metabolic pool, has also been investigated for its supportive role in clinical nutrition and as an adjunct in cancer-related studies, although its application remains context-dependent [50]. It should be noted that this essential amino acid profile remains incomplete, given that lysine was not measured and valine was not detected. Nevertheless, the overall abundant amino acid composition observed in the optimized FVca hydrolysate highlights its potential value as a functional ingredient for plant-based and sustainable nutritional formulations [51,52]. Analysis of the peptide profile of *F. vesiculosus* FVca hydrolysate showed significant peaks in the lower molecular weight fractions particularly within the 3–5 kDa and 5–10 kDa ranges. These fractions are commonly associated with short peptides that have potential biological activities higher than those of larger protein fragments [53,54]. Comparable peptide distributions were obtained for aqueous extracts of *F. vesiculosus* derived from different preparation formats: freshly harvested, commercially available whole-dry algae, and an encapsulated food supplement [55]. The authors aimed to examine the influence of different preparation methods on the chemical composition and bioactivity of *F. vesiculosus,* identifying low-molecular-weight peptides as a key constituent of the hydrolysates [55]. Furthermore, these peptide-rich extracts demonstrated antioxidant, enzyme inhibition and cholesterol permeation modulating activities [55]. Low-molecular-weight peptides are known to display improved gastrointestinal absorption and increased biological efficacy, including stronger antioxidant and immunomodulatory effects, relative to larger peptides [53,54,56,57]. The enrichment of such peptides observed in the optimized FVca hydrolysate therefore supports its potential as a source of functionally relevant peptide fractions, while also highlighting the importance of controlled enzymatic hydrolysis in tailoring peptide size distribution. However, it is important to note that because the reaction mixture was halted by boiling and subsequently freeze-dried in its entirety, the final extract contains denatured cellulase and alcalase proteins. As proteins and peptides exhibit inherent reactivity in Folin–Ciocalteu, ORAC, and ABTS assays, the gravimetric yield, amino acid profiles, and total antioxidant readouts reported here include contributions from these enzyme-derived materials. Therefore, the observed increases in bioactivity under maximum enzyme loading conditions represent the combined properties of the released algal compounds and the residual denatured enzymes.

Regarding the in vitro safety screening on Caco-2 cells, the FVca hydrolysate presented an apparent metabolic inhibition of approximately 60% at the highest concentration tested (10 mg/mL). However, a biphasic profile was observed at concentrations between 0.15 and 5 mg/mL, characterized by negative inhibition values that peaked at approximately −250% at 2.5 mg/mL. Rather than indicating an exponential increase in cellular proliferation, this signal amplification is indicative of a potential chemical interference with the assay methodology. The PrestoBlue assay relies on the conversion of weakly fluorescent resazurin to highly fluorescent resorufin by the reducing environment of metabolically active cells. As previously discussed, the *Fucus vesiculosus* FVca extracts obtained are characterized by a high content of potent antioxidant and reducing compounds, such as phlorotannins and other phenolic compounds. Therefore, it is probable that these bioactive compounds directly reduced the resazurin reagent in an abiotic manner [58,59]. This chemical reduction generates a false-positive fluorescent signal that effectively masks the true cell viability at these intermediate concentrations [58,59]. This phenomenon supports a methodological limitation and a common artifact when evaluating highly reductive marine macroalgae extracts using resazurin- or tetrazolium-based fluorometric and colorimetric assays already discussed by other authors, highlighting the necessity for orthogonal, non-redox viability testing (e.g., LDH release) in future studies. [58,59]. The cytotoxicity of *F. vesiculosus* has been widely studied, particularly due to its rich content of fucoidan, phlorotannins, and other polyphenolic compounds with potential anticancer and therapeutic properties [3,60]. *Fucus vesiculosus* phlorotannin-rich fractions showed potent cytotoxicity against gastrointestinal tumors in Caco-2 (colon) and MKN-28 (gastric) cancer models with minimal effects on healthy cells [12]. Furthermore, fucoidan inhibited ovarian cancer cell growth and angiogenesis (ES-2 and OV-90 ovarian cancer cell lines; zebrafish xenograft cancer model), inducing apoptosis via the endoplasmic reticulum and oxidative stress [61]. These findings highlight the importance of dose control and support the potential of *F. vesiculosus* hydrolysates as functional ingredients, while emphasizing the need for further in vivo safety and bioavailability studies.

## 4. Materials and Methods

### 4.1. Materials

*Fucus vesiculosus* was obtained from land-based, fully controlled cultivation systems under the Integrated Multi-Trophic Aquaculture sustainable concept and was provided by ALGAplus^®^ (Aveiro, Portugal) (dried powder with particle size less than 1.0 mm). The enzymes used for macroalgae hydrolysis were cellulase from *Trichoderma* sp. and alcalase (protease from Sigma-Aldrich, St. Louis, MO, USA). Coomassie (Bradford) Protein Assay Kit was obtained from Thermo Fisher Scientific (Waltham, MA, USA). Gallic acid was obtained from LabChem (Zelienople, PA, USA). Folin & Ciocalteu’s phenol reagent, fluorescein, 2,2′-azo-bis-(2-methylpropionamidine)-dihydrochloride (≥97%), 6-hydroxy-2,5,7,8-tetramethylbroman-2-carboxylic acid (Trolox) (≥97%), 2,2′-Azino-bis(3-ethylbenzothiazoline-6-sulfonic acid) diammonium salt (ABTS), absolute ethanol (CH3CH2OH) and 3,5-Dinitrosalicylic acid (DNS) were obtained from Sigma-Aldrich (St. Louis, MO, USA). Human Caucasian colon carcinoma epithelial cells (Caco-2, ECACC 86010202) were obtained from the European Collection of Authenticated Cell Cultures. Caco-2 cells were cultured in DMEM (4.5 g/L glucose, L-glutamine, without pyruvate), supplemented with 10% (*v*/*v*) fetal bovine serum, 1% (*v*/*v*) penicillin–streptomycin–fungizone, and 1% (*v*/*v*) non-essential amino acids. Cells were incubated at 37 °C in a humid atmosphere containing 95% air and 5% CO_2_. DMEM and NEAA were obtained from Gibco, (Thermo Scientific, Waltham, MA, USA); FBS (Biowest, Nuaillé, France) and penicillin–streptomycin–fungizone were from Lonza (Basel, Switzerland).

### 4.2. Hydrolysis Procedures

Enzyme-assisted hydrolysis was performed according to the procedure described by Nova et al., (2024) [16]. Briefly, 2 g of dried seaweed were added to 50 mL of ultrapure water. The pH was adjusted to 5.0 for cellulase optimal conditions. For cellulase + alcalase enzyme combinations, the pH was initially adjusted for cellulase optimal conditions (5.0), the respective enzyme was added, and the mixture was incubated for half of the incubation time (1.5, 3 or 4.5 h). Subsequently, the pH was adjusted for alcalase optimal conditions (8.0), the alcalase was added, and the resulting mixture was incubated for the remaining incubation time. Enzyme loading in the Design of Experiments (DOE) was calculated strictly relative to the dry weight of the algal biomass (2 g) rather than the reaction volume. Cellulase concentration was expressed as a mass percentage (% *w*/*w*), whereas alcalase was expressed as a volume percentage (% *v*/*w*). For example, an enzyme loading of 3.75% on a dry substrate basis corresponds to the addition of 0.075 g (75 mg) of cellulase and 0.075 mL (75 µL) of alcalase per extraction. When suspended in the standard 50 mL reaction mixture, the 3.75% cellulase loading mathematically yields a final working concentration of 1.5 mg/mL. The enzymes were added to the mixture according to the DOE matrix (Table 11) and incubated at 45, 50, or 55 °C in an orbital shaker for 3, 6, or 9 h in the dark at 125 rpm. After hydrolysis, the reaction was terminated by boiling the samples at 100 °C for 10 min, followed by immediate cooling on ice. The resulting hydrolysate was centrifuged at 5000× *g* for 10 min at 4 °C, and the supernatant was collected. The obtained extract was freeze-dried and stored at −20 °C until further analysis.

### 4.3. Experimental Design

In previous studies, we evaluated the chemical composition and antioxidant capacity of enzyme-assisted extraction of *F. vesiculosus* using alcalase^®^, cellulase^®^, and Viscozyme^®^ in isolated or sequential modes, selecting the best extraction methodologies [16]. To define the key factors influencing the production of hydrolysates enriched in bioactive molecules with high antioxidant activity, a Box–Behnken experimental design was used for enzyme-assisted extraction of *F. vesiculosus* using cellulase alone and in combination with alcalase. Three factors were evaluated for enzyme-assisted extraction with cellulase: hydrolysis temperature (°C) (X_A_), incubation time (hours) (X_B_) and cellulase (%) (X_C_) and four factors for enzyme-assisted extraction with cellulase + alcalase combination: temperature (°C) (X_A_), incubation time (hours) (X_B_), cellulase (%) (X_C_) and alcalase (%) (X_D_). The established levels of the factors were coded as −1 (low), 0 (central) and +1 (high) (Table 10). The DOE resulted in 15 experimental runs for *F. vesiculosus* extraction using cellulase and 27 experimental runs using the cellulase + alcalase combination. Both designs included three center points. Hydrolysis was performed as described in Section 4.2.

### 4.4. Statistical Analysis and Statistical Model

Design of Experiments (DOE) matrices and optimization analyses were performed using Statgraphics Centurion 16^®^ software version 16.1.03. Hydrolysis efficiency comparisons regarding several parameters were analyzed with a significance level of 5% (*p* < 0.05). For the optimization process, full second-order polynomial models were fitted and retained for all responses to maintain hierarchical integrity, with the data fitted to the following equation:

Y = β0 + βAXA + βBXB + βCXC + βDXD + βA,BXAXB + βA,CXAXC + βA,DXAXD + βB,CXBXC + βB,DXBXD + βC,DXCXD + βA,AXA2 + βB,BXB2 + βC,CXC2 + βD,DXD2 + ε(9)

For the Design of Experiments (DOE) matrices, each experimental condition was performed as a single extraction procedure, with the resulting assays measured in triplicate (technical replicates, *n* = 3). To evaluate the pure error and lack-of-fit, the center points of the designs were performed as independent replicates. For the validation of the final optimal FVca condition, the extraction process was performed in triplicate, with each independent extract assayed in triplicate (total *n* = 9). The results in the optimization matrices (Table 1 and Table 3) are expressed as the mean ± standard deviation of the technical replicates.

### 4.5. Extraction Yield

The total extraction yield for each *F. vesiculosus* extract was determined according to the following equation:

***Extraction yield***** (%) = ***** m*****1**/***m*****0  × 100** (10)

where *m*1 is the total mass of the freeze-dried *F. vesiculosus* extract, and *m*0 is the initial mass of dried *F. vesiculosus* used in each extraction.

### 4.6. Folin–Ciocalteu-Derived Total Phenolic Content

Folin–Ciocalteu total phenolic content method was performed as previously described by Rodrigues, Sousa et al., 2015, using gallic acid as the standard (0.025–0.200 mg/mL final concentration in the wells) [62]. Results were expressed as milligrams of gallic acid equivalents per gram of dry seaweed extract (mg GAE/g extract). The Folin–Ciocalteu-derived total phenolic content was determined by colorimetry at 720 nm using a Synergy H1 (BioTek Instruments, Winooski, VT, USA).

### 4.7. Total Antioxidant Capacity

The total antioxidant capacity of the extract solutions was also measured according to the ABTS method described by Gião et al., (2007) [63]. A total of 2 mL of diluted ABTS solution was added to 120 μL of the extract, and the absorbance at 734 nm (Synergy H1; BioTek Instruments, Winooski, VT, USA) was measured using Trolox as the standard (25–250 μM, final concentration in well). Results were expressed as μmol TE/g dry seaweed extract.

### 4.8. Oxygen Radical Absorbance Capacity (ORAC)

The oxygen radical absorbance capacity (ORAC) assay was performed in a black polystyrene 96-well microplate (Nunc, Roskilde, Denmark) according to the method described by [16]. The experiment was carried out in a 75 mM phosphate buffer (pH 7.4). The final reaction mixture was 200 μL—seaweed extract (20 μL) and fluorescein (120 μL) at 70 nM—and the solutions were placed in the well of the microplate. The mixture was preincubated for 10 min at 37 °C. An AAPH solution (60 μL; 12 mM) was then added rapidly. The microplate was immediately placed in the reader, and fluorescence (excitation of 485 nm and emission of 528 nm) was recorded every 1 min for a total of 80 min. Trolox (1–8 μM) was used as the standard for the calibration curve. A blank (fluorescein + AAPH) was performed using the phosphate buffer instead of the antioxidant solution. The microplate was automatically shaken before each reading. Trolox and AAPH solutions were prepared daily, and fluorescein was diluted from a stock solution (1.17 mM) in the same phosphate buffer. Antioxidant curves (fluorescence versus time) were first normalized to the corresponding blank curve for the same assay by multiplying the original data by the factor fluorescence blank, t = 0/fluorescence control, t = 0. From the normalized curves, the area under the fluorescence decay curve (AUC) was calculated using the trapezoidal method. The final AUC values were obtained by subtracting the blank AUC from each result. Regression equations between net AUC and antioxidant concentration were then calculated. Results were expressed in μmol TE/g dry seaweed extract.

### 4.9. Peptide Profile

The peptide profile was analyzed according to the method described by Fernandez Cunha et al. (2023) [64]. The *F. vesiculosus* extract samples were centrifuged at 14,500 rpm for 5 min and filtered through a 22 µm filter. A total volume of 300 µL of each sample was loaded onto two size exclusion chromatography (SEC) columns, Superdex Peptide 10/300 GL and Superdex 200 Increase 10/300 GL, connected in series, using an ÄKTA pure chromatography system (all from GE Healthcare Life Sciences, Freiburg im Breisgau, Germany). Ultrapure water was used as the eluent for the columns. The peaks were detected at 280 nm. The elution flow rate was set at 0.5 mL/min, and fractions of 5 mL were collected starting from the time of sample loading on the column.

### 4.10. HPLC Phenolic Compound Profile

The qualitative and quantitative profiles of phenolic compounds were determined using a Waters Liquid Chromatograph (Waters Series 600, Milford, MA, USA). Chromatographic separation was achieved using an Alltech Adsorbosil C18 reversed-phase column (250 × 4.6 mm i.d., 5 µm particle size, 125 Å pore size; Alltech Associates, Deerfield, IL, USA) equipped with a Symmetry^®^ C18 guard column (Waters Corporation, Milford, MA, USA). This protocol was conducted according to the method described by Coelho et al., 2022 with slight modifications [65]. The mobile phase consisted of two solvents: Solvent A, comprising 100% acetonitrile with 0.2% trifluoroacetic acid (TFA), and Solvent B, comprising a mixture of acetonitrile and water (5:95 *v*/*v*) with 0.2% TFA. The photodiode array (PDA) acquisition wavelength was set between 216 and 600 nm to monitor the maximum absorption of the analytes. Compound identification and quantification were performed using standard solutions of phloroglucinol, gallic acid, epigallocatechin, protocatechuic acid, tiliroside, and p-coumaric acid. Calibration curves were constructed using detection wavelengths of 280 nm for phenolic acids and flavanols, and 320 nm for hydroxycinnamic acids and flavanols, over a concentration range of 0.10 to 100.00 mg/L. The chromatographic separation was achieved using a gradient elution program, as follows: 0–5 min, 100% B; 5–15 min, 100% to 90% B; 15–30 min, 90% to 80% B; 30–45 min, 80% to 70% B; 45–55 min, 70% to 50% B; and 55–58 min, 50% to 0% B. This was followed by a 7 min re-equilibration period at 100% B. All samples were analyzed in triplicate, and results were expressed as milligrams per gram of sample (mg/g sample).

### 4.11. HPLC Amino Acid Profile

Amino acid extraction was performed by acid hydrolysis according to the procedure described by Martins et al., 2024 [66]. Approximately 10 mg of the solid residue sample was mixed with 3 mL of 6 M HCl in a solid-phase micro-extraction (SPME) vial. The mixture was vortexed and purged with nitrogen for 4 min to remove oxygen. The vial was sealed and incubated in an oven at 115 °C overnight to ensure complete hydrolysis. After cooling to room temperature, 4 mL of ultrapure water (Milli-Q) was added, and the pH was adjusted to 3.5 using 10 M NaOH. The final volume was adjusted to 10 mL with ultrapure water, and the solution was filtered through a 0.45 µm filter prior to analysis. Qualitative and quantitative analysis of amino acids was conducted using an Agilent 1200 series HPLC system (Agilent Technologies, Santa Clara, CA, USA) equipped with an LC-20AB solvent delivery unit, a SIL-20A autosampler, a CTO-20A column oven set at 25 °C, and a G1321A fluorescence detector (FLD). The separation was achieved using an Agilent Poroshell HPH-C18 column (2.1 × 200 mm, 5 µm; Agilent Technologies, Santa Clara, CA, USA). Fluorescence detection was monitored with excitation at 356 nm and emission at 445 nm. The mobile phase A consisted of 10 mM Na_2_HPO_4_, 10 mM Na_2_B_4_O_7_, and 5 mM NaN_3_, adjusted to pH 8.2 with concentrated HCl. Mobile phase B was a mixture of acetonitrile, methanol, and water (45:45:10 *v*/*v*/*v*). The elution program utilized a linear gradient starting from 90% mobile phase A to 50% mobile phase A over 43 min. Subsequently, mobile phase B was increased to 100% from 47 to 49 min, before returning to initial conditions (90% A) by 50 min. The flow rate was maintained at 0.9 mL/min. Identification and quantification were performed using standard solutions of alanine, arginine, aspartic acid, cysteine, glutamic acid, glycine, histidine, isoleucine, leucine, methionine, phenylalanine, proline, serine, threonine, tyrosine, and valine. Due to the 6 M HCl hydrolysis conditions, glutamine and asparagine were quantified together with their respective acids as Glx and Asx, and tryptophan was not quantified (Sigma Chemical Co., St. Louis, MO, USA).

### 4.12. Cytotoxicity

The cytotoxicity evaluation was performed according to the ISO 10993-5:2009 standard, as described by Costa et al. (2023) [67]. Briefly, Caco-2 cells (human colorectal adenocarcinoma cell line) were grown to 80–90% confluence. Cells were then detached using TrypLE Express (ThermoScientific, Waltham, MA, USA) and seeded into 96-well microplates at a density of 1 × 10^4^ cells/well. After 24 h, the culture medium was replaced with medium containing various concentrations of algae extracts: 0.15, 0.3, 0.6, 1.25, 2.5, 5 and 10 mg/mL. Dimethyl sulfoxide (DMSO) at 30% (*v*/*v*) was used as the control for cell death, and cells grown only with culture medium were used as the positive control for cell growth. After a 24 h incubation period, PrestoBlue reagent (Thermo Fisher, Waltham, MA, USA) was added to each well, and the plate was incubated for an additional hour. The fluorescence was measured with an excitation at 560 nm and emission at 590 nm using a microplate reader (Synergy H1, Biotek Instruments, Winooski, VT, USA). Results were reported as the percentage of metabolic activity inhibition relative to positive control (untreated cells). All assays were performed in quadruplicate.

## 5. Conclusions

In this work, a sustainable enzyme-assisted methodology was developed to efficiently obtain *F. vesiculosus* hydrolysates with enhanced nutritional and bioactive properties. The application of Design of Experiments enabled the identification of key processing variables affecting hydrolysate yield and antioxidant responses, as well as the determination of optimal conditions for both single-enzyme (cellulase) and dual-enzyme (cellulase + alcalase) hydrolysis strategies. Overall, the combined cellulase–alcalase approach consistently outperformed cellulase alone, yielding higher extraction efficiency and improved antioxidant capacity for all evaluated responses. The FVca hydrolysate with the highest yield and bioactivity (FVca) was selected for further characterization, showing a rich amino acid composition, including several essential amino acids, a predominance of low-molecular-weight peptides associated with biological activity, and a diverse phenolic compound composition led by phloroglucinol (6.23 mg/g). In addition, the FVca hydrolysate demonstrated severe abiotic interference with the redox viability assay at 10 mg/mL in Caco-2 human colorectal adenocarcinoma cell cultures. Overall, the results show the effectiveness of combined enzymatic hydrolysis as a tailored strategy for valorizing *F. vesiculosus* biomass. The resulting hydrolysates show promising functionality, supporting their potential application as bioactive ingredients in food, nutraceutical and biotechnological contexts, and highlighting the relevance of controlled enzymatic processing for sustainable marine biomass exploitation.

## Figures and Tables

**Table 1:** Box–Behnken factorial design matrix for *F. vesiculosus* extraction with cellulase with three factors and four responses.

Run	Factors			Responses			
	Hydrolysis Temperature (°C) (X_A_)	Incubation Time (h) (X_B_)	% Cellulase (X_C_)	Yield (%)	Total Antioxidant Capacity (ABTS) (µmol TE/g Dry Extract)	Folin–Ciocalteu-Derived Total Phenolic Content (mg GAE/g Dry Extract)	ORAC (µmol TE/g Dry Extract)
1	55	6	1.5	29.68	94.34 ± 0.08	250.36 ± 2.71	70.15 ± 0.22
2	55	9	3.75	32.90	86.31 ± 1.07	250.86 ± 1.81	71.86 ± 0.41
**3**	**50**	**6**	**3.75**	**29.64**	**106.80 ± 0.91**	**241.10 ± 0.73**	**226.70 ± 3.73**
4	50	3	1.5	27.83	109.47 ± 1.53	262.98 ± 0.97	262.63 ± 5.21
5	50	9	6	31.99	81.78 ± 0.06	259.55 ± 1.21	205.93 ± 2.32
6	45	3	3.75	30.85	84.77 ± 0.73	234.27 ± 3.05	110.63 ± 1.43
7	50	9	1.5	28.55	101.06 ± 0.84	239.89 ± 0.09	243.10 ± 3.21
8	50	3	6	29.65	88.18 ± 0.21	208.79 ± 1.43	170.79 ± 2.71
**9**	**50**	**6**	**3.75**	**30.23**	**109.50 ± 1.41**	**253.88 ± 2.83**	**215.84 ± 2.52**
10	55	3	3.75	32.80	73.60 ± 1.21	216.28 ± 1.21	76.72 ± 0.43
11	55	6	6	34.82	76.80 ± 0.75	216.87 ± 0.89	70.48 ± 0.31
12	45	6	6	31.23	87.60 ± 0.09	232.64 ± 0.91	72.57 ± 0.77
13	45	9	3.75	29.82	83.90 ± 1.13	224.88 ± 1.89	108.28 ± 0.94
14	45	6	1.5	29.33	101.83 ± 1.04	221.56 ± 2.12	99.18 ± 0.71
**15**	**50**	**6**	**3.75**	**30.15**	**110.82 ± 2.11**	**249.88 ± 1.21**	**232.37 ± 2.32**

**Table 2:** Summary of model fit statistics for the studied responses.

Model	Model *p*-Value	R^2^	Adjusted R^2^	Standard Error
Yield	0.013	0.942	0.838	0.74
FC-derived TPC	0.0022	0.973	0.926	4.64
ABTS	0.0108	0.946	0.850	4.86
ORAC	0.0013	0.978	0.938	18.54

**Table 3:** Box–Behnken factorial design matrix for *F. vesiculosus* extraction with cellulase and alcalase with four factors and four responses.

Run	Factors				Responses			
	Hydrolysis Temperature (°C) (X_A_)	Incubation Time (h) (X_B_)	% Cellulase (X_C_)	% Protease (X_D_)	Yield (%)	ABTS (µmol TE/g Dry Extract)	Folin–Ciocalteu-Derived Total Phenolic Content (GAE/g Dry Extract)	ORAC (µmol TE/g Dry Extract)
1	45	6	3.75	6	34.52	117.8 ± 3.7	221.84 ± 3.7	420.7 ± 7.2
2	45	9	3.75	3.75	35.40	118.1 ± 2.9	225.34 ± 8.3	410.9 ± 4.1
3	50	9	1.5	3.75	35.90	119.2 ± 5.4	222.72 ± 3.2	412.1 ± 11.3
4	45	6	1.5	3.75	34.72	117.9 ± 2.4	222.63 ± 3.2	405.8 ± 8.2
**5**	**50**	**6**	**3.75**	**3.75**	**34.25**	**118.3 ± 1.5**	**232.58 ± 7.4**	**410.8 ± 7.5**
6	50	6	1.5	6	33.63	114.4 ± 4.2	215.7 ± 2.1	410.8 ± 3.3
7	50	6	6	1.5	35.30	120.4 ± 5.8	226.31 ± 5.3	423.5 ± 7.3
8	45	3	3.75	3.75	32.92	106.1 ± 2.1	215.6 ± 3.1	396.9 ± 2.5
9	55	6	1.5	3.75	34.71	117.7 ± 7.2	232.43 ± 2.2	394.8 ± 3.1
10	50	6	6	6	39.24	145.4 ± 3.6	252.38 ± 1.5	475.2 ± 4.3
**11**	**50**	**6**	**3.75**	**3.75**	**34.33**	**115.6 ± 1.7**	**232.7 ± 2.9**	**402.3 ± 4.6**
12	55	6	3.75	1.5	37.02	127.2 ± 4.8	246.7 ± 3.1	421.2 ± 1.2
13	50	9	3.75	1.5	34.23	120.3 ± 2.3	237.16 ± 2.3	413.2 ± 6.3
14	55	6	3.75	6	38.70	131.7 ± 5.1	242.13 ± 9.8	463.9 ± 8.6
15	45	6	6	3.75	35.60	119.6 ± 4.7	238.09 ± 4.1	415.5 ± 2.4
16	50	3	3.75	1.5	33.23	116.4 ± 3.1	214.75 ± 8.2	441.5 ± 4.1
17	45	6	3.75	1.5	34.67	122.9 ± 6.8	230.7 ± 7.3	422.6 ± 1.6
18	50	9	6	3.75	37.93	135.9 ± 5.5	247.61 ± 5.1	435.7 ± 2.3
19	50	3	1.5	3.75	33.50	116.8 ± 6.1	218.8 ± 8.3	411.1 ± 4.3
20	55	9	3.75	3.75	35.83	117.1 ± 1.5	232.24 ± 9.1	443.3 ± 2.5
21	55	6	6	3.75	38.35	138.6 ± 2.4	247.86 ± 13.1	454.6 ± 1.4
22	50	3	6	3.75	36.11	120.7 ± 3.6	238.49 ± 5.1	451.7 ± 5.1
23	50	6	1.5	1.5	34.70	127.1 ± 3.7	236.16 ± 6.4	427.5 ± 1.5
24	50	3	3.75	6	34.73	125.0 ± 2.9	230.16 ± 3.1	426.8 ± 2.6
**25**	**50**	**6**	**3.75**	**3.75**	**34.23**	**124.6 ± 3.0**	**228.26 ± 1.5**	**420.7 ± 1.5**
26	55	3	3.75	3.75	35.68	129.9 ± 3.1	223.13 ± 4.6	425.5 ± 4.3
27	50	9	3.75	6	36.89	135.7 ± 7.1	218.54 ± 1.8	440.2 ± 5.1

**Table 4:** Summary of model fit statistics for the studied responses.

Model	Model *p*-Value	R^2^	Adjusted R^2^	Standard Error
Yield	0.0003	0.907	0.804	0.74
FC-derived TPC	0.0005	0.902	0.787	4.91
ABTS	0.0004	0.904	0.792	3.94
ORAC	0.0003	0.911	0.807	8.81

**Table 5:** Single and multiple response optimization for *F. vesiculosus* extracted with cellulase.

Factors	Response				Multiple Responses
	Yield (%)	Total Antioxidant Capacity (ABTS) (µmol TE/g Dry Extract)	Folin–Ciocalteu-Derived Total Phenolic Content (mg GAE/g Dry Extract)	ORAC (µmol TE/g Dry Extract)	
Temperature (°C)	55.0	49.6	50.5	49.7	50.9
Incubation Time (h)	9.0	5.8	3.0	3.0	7.8
Cellulase (%)	6.0	1.5	1.5	1.5	4.5

**Table 6:** Single and multiple response optimization for *F. vesiculosus* extracted with cellulase and alcalase.

Factors	Response				Multiple Responses
	Yield (%)	Total Antioxidant Capacity (ABTS) (µmol TE/g Dry Extract)	Folin–Ciocalteu-Derived Total Phenolic Content (mg GAE/g Dry Extract)	ORAC (µmol TE/g Dry Extract)	
Temperature (°C)	55.0	54.7	55.0	55.0	53.81
Incubation Time (h)	5.1	9.0	5.0	9.0	5.0
Cellulase (%)	5.98	5.97	6.0	5.99	5.97
Alcalase (%)	6.0	6.0	6.0	5.99	5.95

**Table 7:** Pearson correlation matrix for the dual-enzyme extraction responses.

Response	Yield	ABTS	FC-Derived TPC	ORAC
Yield	1.000	0.852	0.745	0.749
ABTS	0.852	1.000	0.654	0.746
FC-derived TPC	0.745	0.654	1.000	0.519
ORAC	0.749	0.746	0.519	1.000

**Table 8:** *Fucus vesiculosus* hydrolysate obtained by combined cellulase and alcalase hydrolysis DOE and experimental results for yield, total antioxidant capacity, Folin–Ciocalteu-derived total phenolic content and ORAC.

Parameters	DOE Results	Experimental Results
Yield (%)	40.6	39.41 ± 3.6
Total antioxidant capacity (µmol TE/g dry extract)	149.0	142.80 ± 5.1
Folin–Ciocalteu-derived total phenolic content (mg GAE/g dry extract)	256.0	252.57 ± 5.3
ORAC (µmol TE/g dry extract)	487.2	477.64 ± 12.8

DOE = Design of Experiment.

**Table 9:** Amino acids profile of *F. vesiculosus* hydrolysate obtained by combined action of cellulase and alcalase hydrolysis.

Amino Acid	Results (mg/g)
Glx	27.73
Asx	10.72
Serine	2.75 ± 0.37
Histidine	2.11 ± 0.42
Glycine	1.25 ± 0.33
Threonine	0.90 ± 0.07
Arginine	3.55 ± 0.54
Alanine	1.52 ± 0.20
Tyrosine	2.64 ± 0.54
Valine	nd
Methionine	1.03 ± 0.17
Phenylalanine	1.70 ± 0.24
Isoleucine	0.34 ± 0.02
Leucine	0.34 ± 0.03

nd = not detected.

**Table 10:** Phenolic compounds profile of *F. vesiculosus* hydrolysate obtained by combined action of cellulase and alcalase hydrolysis.

Compound	RT (min)	λmax (nm)	Concentration (mg/g Sample)
Phloroglucinol	2.53	279	6.23
Gallic acid	3.80	-	1.21
Unidentified	6.00	223, 279	-
Epigallocatechin	7.55	266	-
Protocatechuic acid	8.46	-	3.37
Unidentified	10.46	223, 275	-
Unidentified	18.43	219, 279	-
Tiliroside	21.01	271	-
*p*-Coumaric acid	33.01	-	3.14
Unidentified	58.00	220, 346	-

**Table 11:** Levels of experimental factors for the experimental designs.

	Factors	Levels		
		Low (−1)	Central (0)	High (+1)
**FV** **C**	Temperature (X_A_)	45	50	55
	Incubation time (X_B_)	3	6	9
	% Cellulase (X_C_)	1.5	3.75	6
**FV** **CA**	Temperature (X_A_)	45	50	55
	Incubation time (X_B_)	3	6	9
	% Cellulase (X_C_)	1.5	3.75	6
	% Protease (X_D_)	1.5	3.75	6

FVC: *F. vesiculosus* extracted with cellulase. FVCA: *F. vesiculosus* extracted with cellulase + alcalase combination.

## Data Availability

All data generated or analyzed during this study are included in this published article.

## Supplementary Materials

The following supporting information can be downloaded at: https://www.mdpi.com/article/10.3390/md24070251/s1, Table S1: FVc analysis of variance (ANOVA) for yield; Table S2: FVc analysis of variance (ANOVA) for Folin–Ciocalteu-derived total phenolic content; Table S3: FVc analysis of variance (ANOVA) for total antioxidant capacity (ABTS); Table S4: FVc analysis of variance (ANOVA) for ORAC; Table S5: FVca analysis of variance (ANOVA) for yield; Table S6: FVca analysis of variance (ANOVA) for Folin–Ciocalteu-derived total phenolic content; Table S7: FVca analysis of variance (ANOVA) for total antioxidant capacity (ABTS); Table S8: FVca analysis of variance (ANOVA) for ORAC.
