# Supplementary material for: Enzymatic Hydrolysates from Fucus vesiculosus: Optimal Process, Chemical Profile and Bioactivity
Source: Mar Drugs. 2026 Jul 18;24(7):251. doi: 10.3390/md24070251 (PMC13412148; doi:10.3390/md24070251)
Supplement: Supplementary file 1 [file marinedrugs-24-00251-s001.zip › Table S1. FVc analysis of variance (ANOVA) for yield..pdf]

Table S1. FVc analysis of variance (ANOVA) for yield.

Model	Sum of Squares	DF	Mean Square	F-Value
A:Temperature	10.0576	1	10.0576	18.36
B:Incubation Time	0.567113	1	0.567113	1.04
C:Cellulase	18.9112	1	18.9112	34.52
AA	10.3335	1	10.3335	18.86
AB	0.319225	1	0.319225	0.58
AC	2.6244	1	2.6244	4.79
BB	0.0280006	1	0.0280006	0.05
BC	0.6561	1	0.6561	1.20
CC	0.634631	1	0.634631	1.16
R^2 = 0.942, Adj-R^2 = 0.838, Standard error = 0.74				
