# Supplementary material for: Enzymatic Hydrolysates from Fucus vesiculosus: Optimal Process, Chemical Profile and Bioactivity
Source: Mar Drugs. 2026 Jul 18;24(7):251. doi: 10.3390/md24070251 (PMC13412148; doi:10.3390/md24070251)
Supplement: Supplementary file 1 [file marinedrugs-24-00251-s001.zip › Table S2. FVc analysis of variance (ANOVA) for Folin-Ciocalteu.pdf]

Table S2. FVc analysis of variance (ANOVA) for Folin-Ciocalteu-derived total phenolic content.

Model	Sum of Squares	DF	Mean Square	F-Value
A:Temperature	55.2301	1	55.2301	2.56
B:Incubation Time	349.272	1	349.272	16.20
C:Cellulase	405.27	1	405.27	18.80
AA	784.853	1	784.853	36.41
AB	483.34	1	483.34	22.42
AC	496.621	1	496.621	23.04
BB	16.8238	1	16.8238	0.78
BC	1363.46	1	1363.46	63.25
CC	41.4266	1	41.4266	1.92
R^2 = 0.973, Adj-R^2 = 0.926, Standard error = 4.64				
