# Supplementary material for: Enzymatic Hydrolysates from Fucus vesiculosus: Optimal Process, Chemical Profile and Bioactivity
Source: Mar Drugs. 2026 Jul 18;24(7):251. doi: 10.3390/md24070251 (PMC13412148; doi:10.3390/md24070251)
Supplement: Supplementary file 1 [file marinedrugs-24-00251-s001.zip › Table S3. FVc analysis of variance (ANOVA) for ABTS.pdf]

Table S3. FVc analysis of variance (ANOVA) for total antioxidant capacity (ABTS).

Model	Sum of Squares	DF	Mean Square	F-Value
A:Temperature	91.8013	1	91.8013	3.89
B:Incubation Time	1.125	1	1.125	0.05
C:Cellulase	653.411	1	653.411	27.70
AA	939.823	1	939.823	39.84
AB	46.24	1	46.24	1.96
AC	2.7225	1	2.7225	0.12
BB	441.034	1	441.034	18.70
BC	1.0	1	1.0	0.04
CC	32.2231	1	32.2231	1.37
R^2 = 0.946, Adj-R^2 = 0.850, Standard error = 4.86				
