# Supplementary material for: Enzymatic Hydrolysates from Fucus vesiculosus: Optimal Process, Chemical Profile and Bioactivity
Source: Mar Drugs. 2026 Jul 18;24(7):251. doi: 10.3390/md24070251 (PMC13412148; doi:10.3390/md24070251)
Supplement: Supplementary file 1 [file marinedrugs-24-00251-s001.zip › Table S4. FVc analysis of variance (ANOVA) for ORAC.pdf]

Table S4. FVc analysis of variance (ANOVA) for ORAC.

Model	Sum of Squares	DF	Mean Square	F-Value
A:Temperature	1287.78	1	1287.78	3.75
B:Incubation Time	9.03125	1	9.03125	0.03
C:Cellulase	3010.88	1	3010,88	8.76
AA	70108.4	1	70108,4	203.95
AB	1.5625	1	1.5625	0.00
AC	182.25	1	182.25	0.53
BB	81.7078	1	81.7078	0.24
BC	745.29	1	745.29	2.17
CC	303.803	1	303.803	0.88
R^2 = 0.978, Adj-R^2 = 0.938, Standard error = 18.54				
