# Supplementary material for: Enzymatic Hydrolysates from Fucus vesiculosus: Optimal Process, Chemical Profile and Bioactivity
Source: Mar Drugs. 2026 Jul 18;24(7):251. doi: 10.3390/md24070251 (PMC13412148; doi:10.3390/md24070251)
Supplement: Supplementary file 1 [file marinedrugs-24-00251-s001.zip › Table S5. FVca analysis of variance (ANOVA) for YIELD.pdf]

Table S5. FVca analysis of variance (ANOVA) for yield.

Model	Sum of Squares	DF	Mean Square	F-Value
A:Temperature	12.9376	1	12.9376	23.65
B:Incubation time	8.35001	1	8.35001	15.26
C:Cellulase	19.6864	1	19.6864	35.99
D:Alcalase	6.10613	1	6.10613	11.16
AA	3.56067	1	3.56067	6.51
AB	1.35722	1	1.35722	2.48
AC	1.9044	1	1.9044	3.48
AD	0.837225	1	0.837225	1.53
BB	0.0489815	1	0.0489815	0.09
BC	0.0841	1	0.0841	0.15
BD	0.3364	1	0.3364	0.61
CC	5.4765	1	5.4765	10.01
CD	6.27502	1	6.27502	11.47
DD	2.32027	1	2.32027	4.24
R^2 = 0.907, Adj-R^2 = 0.804, Standard error = 0.74				
