# Supplementary material for: Enzymatic Hydrolysates from Fucus vesiculosus: Optimal Process, Chemical Profile and Bioactivity
Source: Mar Drugs. 2026 Jul 18;24(7):251. doi: 10.3390/md24070251 (PMC13412148; doi:10.3390/md24070251)
Supplement: Supplementary file 1 [file marinedrugs-24-00251-s001.zip › Table S6. FVca analysis of variance (ANOVA) for Folin-Ciocalteu.pdf]

Table S6. FVca analysis of variance (ANOVA) for Folin-Ciocalteu-derived total phenolic content.

Model	Sum of Squares	DF	Mean Square	F-Value
A:Temperature	411.724	1	411.724	17.06
B:Incubation time	151.799	1	151.799	6.29
C:Cellulase	872.108	1	872.108	36.13
D:Alcalase	10.1384	1	10.1384	0.42
AA	5.53068	1	5.53068	0.23
AB	0.099225	1	0.099225	0
AC	0.000225	1	0.000225	0
AD	4.60103	1	4.60103	0.19
BB	176.359	1	176.359	7.31
BC	6.76	1	6.76	0.28
BD	289.51	1	289.51	11.99
CC	68.3382	1	68.3382	2.83
CD	541.26	1	541.26	22.42
DD	0.328904	1	0.328904	0.01
R^2 = 0.902, Adj-R^2 = 0.787, Standard error = 4.91				
