# Supplementary material for: Enzymatic Hydrolysates from Fucus vesiculosus: Optimal Process, Chemical Profile and Bioactivity
Source: Mar Drugs. 2026 Jul 18;24(7):251. doi: 10.3390/md24070251 (PMC13412148; doi:10.3390/md24070251)
Supplement: Supplementary file 1 [file marinedrugs-24-00251-s001.zip › Table S7. FVca analysis of variance (ANOVA) for total antioxidant capacity (ABTS)..pdf]

Table S7. FVca analysis of variance (ANOVA) for total antioxidant capacity (ABTS).

Model	Sum of Squares	DF	Mean Square	F-Value
A:Temperature	298.003	1	298.003	19.17
B:Incubation Time	82.1633	1	82.1633	5.29
C:Cellulase	379.688	1	379.688	24.43
D:Alcalase	106.207	1	106.207	6.83
AA	0.0408333	1	0.0408333	0
AB	153.76	1	153.76	9.89
AC	92.16	1	92.16	5.93
AD	23.04	1	23.04	1.48
BB	1.40083	1	1.40083	0.09
BC	40.96	1	40.96	2.64
BD	11.56	1	11.56	0.74
CC	67.2133	1	67.2133	4.32
CD	355.322	1	355.322	22.86
DD	126.75	1	126.75	8.15

R^2 = 0.904, Adj-R^2 = 0.792, Standard error = 3.94
