# Supplementary material for: Enzymatic Hydrolysates from Fucus vesiculosus: Optimal Process, Chemical Profile and Bioactivity
Source: Mar Drugs. 2026 Jul 18;24(7):251. doi: 10.3390/md24070251 (PMC13412148; doi:10.3390/md24070251)
Supplement: Supplementary file 1 [file marinedrugs-24-00251-s001.zip › Table S8. FVca analysis of variance (ANOVA) for ORAC..pdf]

Table S8. FVca analysis of variance (ANOVA) for ORAC.

Model	Sum of Squares	DF	Mean Square	F-Value
A:Temperature	1427.9	1	1427.9	18.42
B:Incubation time	0.300833	1	0.300833	0
C:Cellulase	3139.57	1	3139.57	40.49
D:Alcalase	646.801	1	646.801	8.34
AA	20.3668	1	20.3668	0.26
AB	3.61	1	3.61	0.05
AC	627.503	1	627.503	8.09
AD	497.29	1	497.29	6.41
BB	198.725	1	198.725	2.56
BC	72.25	1	72.25	0.93
BD	434.722	1	434.722	5.61
CC	282.593	1	282.593	3.64
CD	1169.64	1	1169.64	15.09
DD	1344.79	1	1344.79	17.34

R^2 = 0.911, Adj-R^2 = 0.807, Standard error = 8.81
